# Expansion of myeloid-derived suppressor cells in patients with severe coronavirus disease (COVID-19)

**DOI:** 10.1038/s41418-020-0572-6

**Published:** 2020-06-08

**Authors:** Chiara Agrati, Alessandra Sacchi, Veronica Bordoni, Eleonora Cimini, Stefania Notari, Germana Grassi, Rita Casetti, Eleonora Tartaglia, Eleonora Lalle, Alessandra D’Abramo, Concetta Castilletti, Luisa Marchioni, Yufang Shi, Andrea Mariano, Jin-Wen Song, Ji-Yuan Zhang, Fu-Sheng Wang, Chao Zhang, Gian Maria Fimia, Maria R. Capobianchi, Mauro Piacentini, Andrea Antinori, Emanuele Nicastri, Markus Maeurer, Alimuddin Zumla, Giuseppe Ippolito

**Affiliations:** 1 National Institute for Infectious Diseases, Lazzaro Spallanzani-IRCCS, Via Portuense, 292-00149, Rome, Italy; 2 Institutes for Translational Medicine, Soochow University Medical College, Suzhou, China; 3 Institute of Health Sciences, Chinese Academy of Sciences, Shanghai, China; 4 Treatment and Research Center for Infectious Diseases, The Fifth Medical Center of PLA General Hospital, National Clinical Research Center for Infectious Diseases, 100039 Beijing, China; 5 Department of Molecular Medicine, University of Rome “Sapienza”, Rome, Italy; 6 Department of Biology, University of Rome “Tor Vergata”, Rome, Italy; 7 Immunotherapy Programme, Champalimaud Centre for the Unknown, Lisbon, Portugal; 8 I Med Clinic, University of Mainz, Mainz, Germany; 9 Division of Infection and Immunity, University College London, London, UK; 10 National Institute or Health Research Biomedical Research Centre, University College London Hospitals NHS Foundation Trust, London, UK

**Keywords:** Infectious diseases, Infectious diseases

## Abstract

SARS-CoV-2 is associated with a 3.4% mortality rate in patients with severe disease. The pathogenesis of severe cases remains unknown. We performed an in-depth prospective analysis of immune and inflammation markers in two patients with severe COVID-19 disease from presentation to convalescence. Peripheral blood from 18 SARS-CoV-2-infected patients, 9 with severe and 9 with mild COVID-19 disease, was obtained at admission and analyzed for T-cell activation profile, myeloid-derived suppressor cells (MDSCs) and cytokine profiles. MDSC functionality was tested in vitro. In four severe and in four mild patients, a longitudinal analysis was performed daily from the day of admission to the early convalescent phase. Early after admission severe patients showed neutrophilia, lymphopenia, increase in effector T cells, a persisting higher expression of CD95 on T cells, higher serum concentration of IL-6 and TGF-β, and a cytotoxic profile of NK and T cells compared with mild patients, suggesting a highly engaged immune response. Massive expansion of MDSCs was observed, up to 90% of total circulating mononuclear cells in patients with severe disease, and up to 25% in the patients with mild disease; the frequency decreasing with recovery. MDSCs suppressed T-cell functions, dampening excessive immune response. MDSCs decline at convalescent phase was associated to a reduction in TGF-β and to an increase of inflammatory cytokines in plasma samples. Substantial expansion of suppressor cells is seen in patients with severe COVID-19. Further studies are required to define their roles in reducing the excessive activation/inflammation, protection, influencing disease progression, potential to serve as biomarkers of disease severity, and new targets for immune and host-directed therapeutic approaches.

## Introduction

The novel coronavirus SARS-CoV-2 global epidemic progresses relentlessly [1]. A range of clinical manifestations of COVID-19 disease occurs, from mild, moderate, severe to fulminant pulmonary disease have been described. SARS-CoV-2 infection is associated with a 3.4% mortality rate. The pathogenesis of SARS-CoV-2 infection remains unknown. Whilst most patients develop mild disease (fever, runny nose, malaise, dry cough and fatigue) with a good prognosis, others develop a severe pneumonia characterized by acute respiratory distress syndrome (ARDS) and multiple organ failure with high rate of intensive unit care admission (ICU) [2–4]. Immunological studies of patients with two other lethal zoonotic coronavirus infections, SARS-CoV and MERS-CoV, suggest underlying immune-based pathogenic mechanisms with lymphopenia with exacerbated dysregulated inflammatory responses that correlate with disease severity [5–7]. Lymphopenia and increase in inflammatory mediators were reported in severe COVID-19 disease [2–8] and a longitudinal study suggested that the neutrophil/lymphocyte ratio could be an important prognostic factor for early identification of severe COVID-19 patients [9]. We performed a prospective study of daily examination of peripheral blood immune activation and inflammation profiles of 18 patients with laboratory confirmed SARS-CoV-2 infection admitted to the INMI in Rome, Italy, 9 with severe COVID-19 disease and 9 with mild disease (from the day of admission to the early convalescent phase).

## Methods

### Ethics and IRB

The research protocol was approved by the local INMI, Rome Ethical Committee and patients provided written informed consent.

### Patients and clinical data

Eighteen patients with COVID-19 disease admitted to the National Institute for Infectious Diseases “L Spallanzani” in Rome were enrolled in the study. The local Ethical Committee approved the study and all patients provided written consent. Nine patients presented a severe disease with fever, cough and ARDS [PaO2 = (partial pressure of arterial oxygen)/FIO2 (Fraction of inspired oxygen) Ratio < 300 mmHg]. Nine patients presented mild symptoms (mild conjunctivitis, axillary temperature of 37.5 °C, or with no clinical sign). All COVID-19 patients were sampled at hospital admission. Four severe (Pt1, Pt2, Pt15, and Pt18) and four mild (Pt3, Pt4, Pt5 and Pt396) patients were longitudinally sampled from admission to convalescent phase. Eight healthy donors (HD) were used as control.

#### Haematological indices

Whole venous blood was drawn daily for full blood counts, differential counts and immunological analysis.

#### Peripheral blood mononuclear cells (PBMCs)

Peripheral venous blood was obtained and PBMC were isolated from peripheral blood by density gradient centrifugation (Lympholyte-H; Cederlane, Canada). PBMCs were resuspended in RPMI 1640 (Corning) supplemented with 10% heat-inactivated foetal bovine serum (EuroClone, Italy), 2 mmol/L L-glutamine, 10 mmol/L HEPES buffer (N-2-hydroxyethylpiperazine-N-2-ethane sulfonic acid), 2 mmol/L penicillin and 50 μg/mL streptomycin.

#### Lymphocytes characterization

T lymphocytes profile was analyzed by staining whole blood using preconfigured customized lyophilized reagent tubes following manufacturer’s procedures (BD Lyotube TM 8-colour CD4 and CD8 bundle, (BD Biosciences San Jose, CA, USA including CD4 and CD8 specific “Lyotubes” code 624746): CD4 Lyotube: CD95 FITC/CCR7 PEl CD3 PerCPCy 5.5/CD25 PE-Cy7/CD127 Alexa Fluor 647/CD45 APC-H7/CD4 AmCyan/CD45RA V450; clones: DX2/150503/SK7/2A3/HIL-7R-M-21/2D1/SK3/HIlOO. CD8 Lyotube: CD38 FITC/CCR7 PEl CD3 PerCPCy 5.5/CD69 PE-Cy7/CDl27 Alexa Fluor 647/CD45 APC-H7/CD8 AmCyan/CD45RA V450; clones: HB7/150503/SK7/L78/HIL-7R-M-21/2D1/SK1/HIlOO.) Data were acquired by FACSCanto II flow cytometer (BD Biosciences). Data analysis was performed using FlowJo 9.3.2 (BD Biosciences).

T- and NK-cell perforin content was analyzed by flow cytometry. HD were used as control. Briefly, 50 µl of whole blood was stained with a surface antibody’s cocktail (anti-CD3 PB cat. 558117, anti-CD56 PeCy7 cat. 557747 from BD Biosciences) for 20 min at 4 °C, lysed and fixed with a buffer (BD PharmLyse) for 15 min at room temperature in the dark and washed with PBS 1×. Finally, cells were labelled with anti-Perforin PE antibody (cat. 563763, BD Biosciences) for 20 min at RT in the dark. After one wash, cells were acquired using a Cytoflex-LX (Bekman-Coulter) and analyzed by CytExpert software (Beckman Coulter).

#### Plasma and culture supernatants cytokine levels

Plasma samples were obtained after speed centrifugation for 10 min at 2000 rpm and immediately stored at −80 °C. Culture supernatants were collected and stored at −80 °C. IL-1β, IL-6, IL-8, TNF-α, IFN-γ and TGFβ were quantified in plasma and supernatants samples by using the automated ELISA assay (ELLA microfluidic analyzer, Protein Simple, Bio-Techne, Minneapolis, USA).

#### Myeloid-derived suppressor cells (MDSC) identification and isolation

MDSC identification was performed on PBMCs isolated from Lithium-Heparin tubes, and stained by using customized Duraclone Tubes (CD11b FITC, HLA-DR ECD, CD14 PC5.5, CD33 PC7, CD80 APC, DRAQ7, CD56 APC-alexa750, CD19 APC-alexa750, CD3 APC-alexa750, CD15 Pacific Blue, CD45KrO, cat. B74697, Beckman Coulter, CA, USA) following manufacturer’s instruction. Polymorphonuclear (PMN) MDSC were purified from PBMC by immunomagnetic sorting by using CD15 microbeads (cat. 130-046-601, Miltenyi Biotec, Germany) following the manufacturer’s procedure. The purity of sorted G-MDSC was >90% as verified by flow cytometry.

#### Proliferation assay

PBMC and MDSC-depleted PBMC were labelled with CFDA-SE (Vibrant CFDA-SE cell tracer kit, Invitrogen) according with manufacturer’s instruction. CFDA-SE-stained PBMC, MDSC-depleted PBMC (depleted) or depleted cells seeded with purified polymorphonuclear-MDSC (PMN-MDSC) (1:5 ratio) were stimulated with Staphylococcus enterotoxin B (SEB, 800 ng/ml, Sigma Aldrich). Cells were maintained at 37 °C in humidified air with 5% CO_2_. After 2 and 4 days, supernatants were collected, and at day 4 cells were stained with CD3 APC (cat. 340661, BD Biosciences) washed and acquired by CytoFlex flow cytometer (Beckman Coulter). Data were analyzed by CytExpert software (Beckman Coulter).

### Statistical analysis

Quantitative variables were compared with nonparametric Mann–Whitney test. A *p* value lower than 0.05 was considered statistically significant. Statistical analyses were performed using GraphPad Prism v8.0 (GraphPad Software, Inc).

## Results

### WBC differential counts

**Fig. 1:**
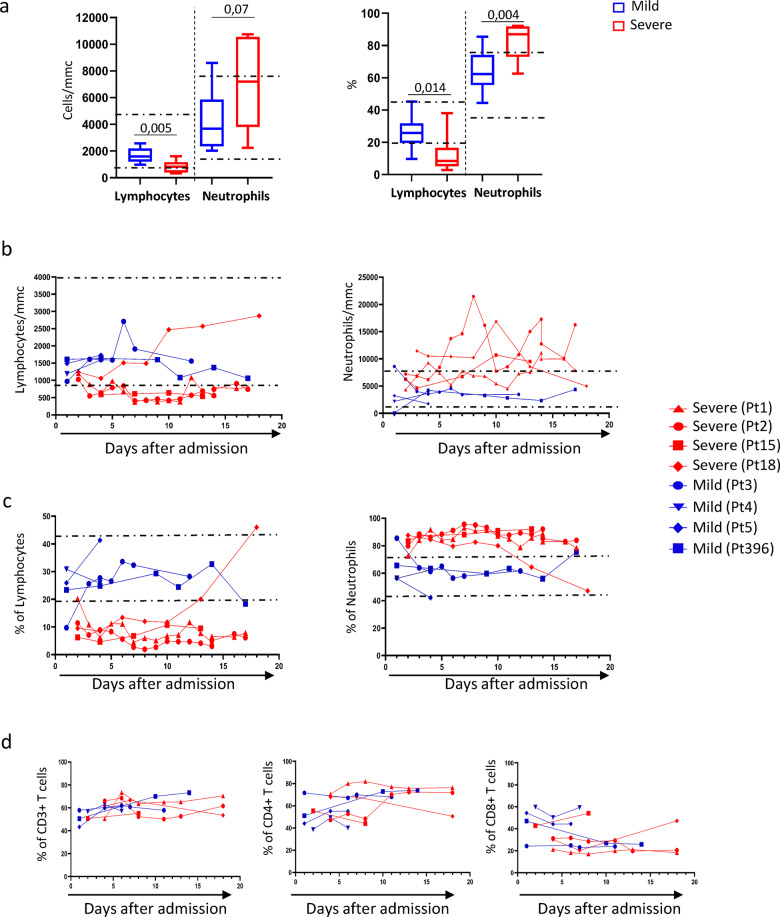
Neutrophils and lymphocytes distribution in SARS-CoV-2-infected patients. **a** Neutrophils/lymphocytes absolute number and percentage were analyzed in nine severe (red boxes) and in nine mild (blue boxes) COVID-19 patients. Results are shown as box and whiskers. The Mann–Whitney test was used. **b** Kinetic analysis of neutrophils/lymphocytes absolute number and percentage in four severe and in four mild COVID-19 patients. **c** Kinetic analysis of CD3+, CD4+ and CD8+ T-cell frequency among T lymphocytes was analyzed by flow cytometry (**d**). Red lines and blue lines represent severe (Pt1 and Pt2, Pt15, Pt18) and mild (Pt3, Pt4, Pt5 and Pt396) SARS-CoV-2-infected patients, respectively. Dashed line: normal values.

We performed the immunological profiling of 18 SARS-CoV-2-infected patients (9 with severe and 9 with mild diseases). At admission, the analysis of WBC count showed a significant lower lymphocytes count (and frequency) and a parallel higher neutrophil count (and frequency) in severe than in mild COVID-19 patients (Fig. 1a, b). Nevertheless, we performed a longitudinal analysis in four severe and in four mild patients in order to analyze the immunological changes during the course of the COVID-19. The kinetic of leucocytes showed an increase in neutrophil count paralleled by an early and rapid decrease of lymphocytes during the course of severe diseases (Fig. 1b). A similar kinetic was also observed for the neutrophils and lymphocytes percentage (Fig. 1c). In contrast, the patients with mild disease quickly exhibited a leucocyte count and frequency within the normal range (Fig. 1b, c). Flow cytometric analysis of T-cell subsets showed no major differences among patients with severe or mild symptoms (Fig. 1d).

### T-cell activation and differentiation profile

**Fig. 2:**
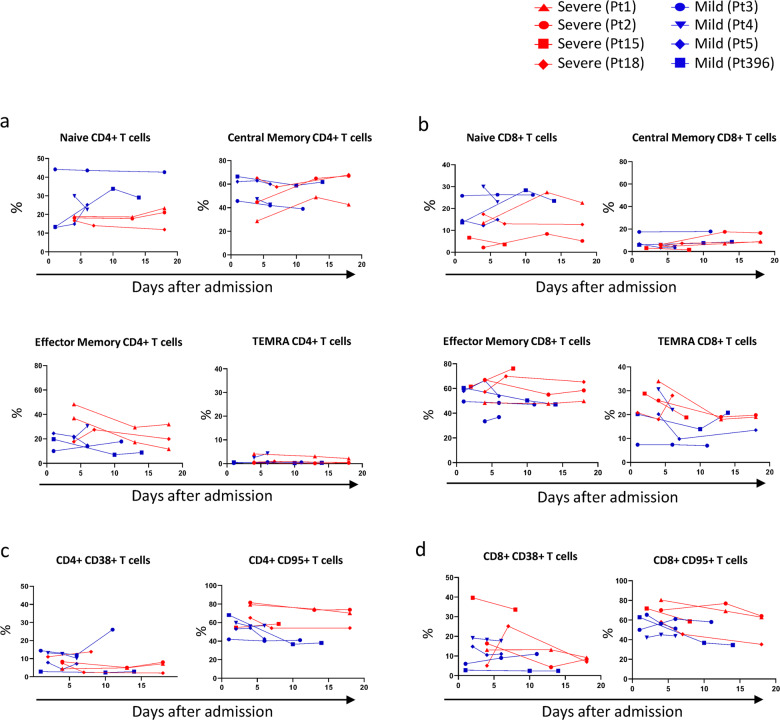
Differentiation and activation profile of CD4+ and CD8+ T lymphocytes in SARS-CoV-2-infected patients. Differentiation profile [Naive, NA/Precursor (CD45RA+CCR7+), Central Memory (CD45RA−CCR7+), Effectory Memory (CD45RA−CCR7−); Terminally differentiated T cell (TEMRA: CD45RA+CCR7−)] in CD4 (**a**) and in CD8 (**b**) T lymphocytes. The frequency of CD38 and CD95 expressing CD4 (**c**) and CD8 (**d**) T cells was analyzed in SARS-CoV-2-infected patients by flow cytometry. Red lines and blue lines represent severe (Pt1, Pt2, Pt15 and Pt18) and mild (Pt3, Pt4, Pt5 and Pt396) SARS-CoV-2-infected patients, respectively. Dashed line: median of normal values.

The differentiation profile and activation markers in CD4 and CD8 T cells showed, during the early phase of the disease, a lower frequency of precursor CD4+ T cells with a parallel higher frequency of effector memory (EM) CD4+ T cells in blood from patients with severe COVID-19 infection (Fig. 2a). A lower frequency of precursor CD8+ T cells was observed in patients with severe disease with a parallel higher frequency of EM and terminally differentiated (TEMRA) CD8+ T cells (Fig. 2b).

**Fig. 3:**
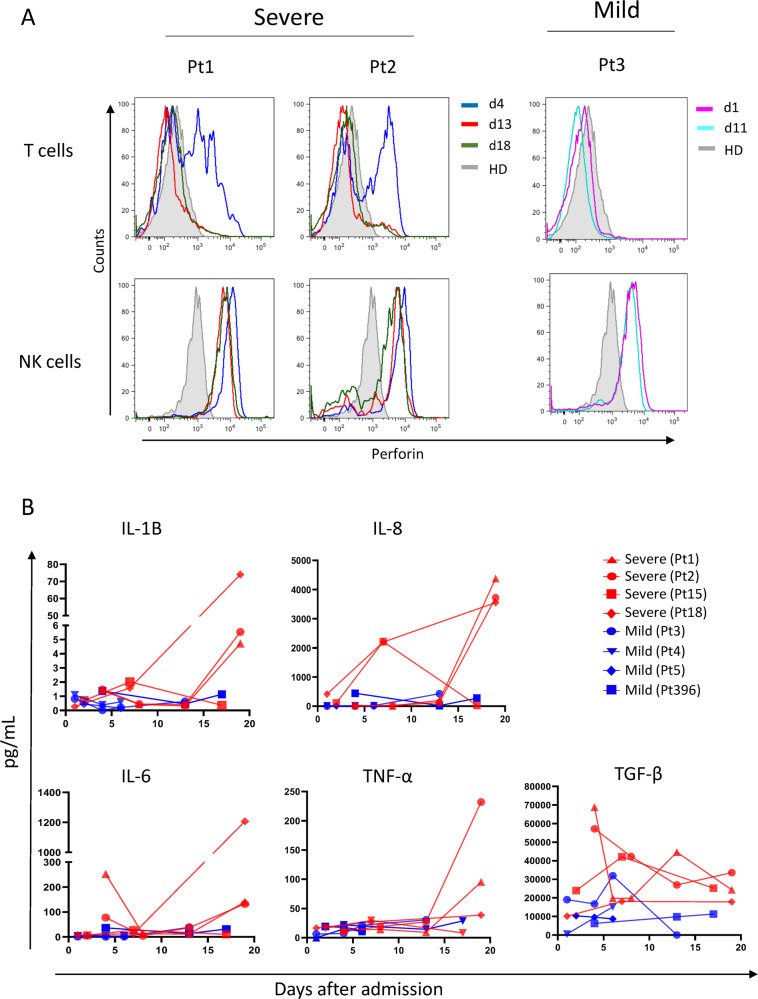
Perforin expression and inflammatory mediators in SARS-CoV-2-infected patients. **a** Perforin in CD3+ T cells and in CD3-Natural Killer cells was analyzed during different time points in severe (day 4, blue line; day 13, red line; day 18, green line) and in mild (day 1, violet line; day 11, blue light line) SARS-CoV-2-infected patients by flow cytometry. Full grey histogram represent healthy donors (HD) (**a**). Pro-inflammatory factors of SARS-CoV-2-infected patients were analyzed by automated ELISA (**b**). Red lines and blue lines represent severe (Pt1, Pt2, Pt15 and Pt18) and mild (Pt3, Pt4, Pt5 and Pt396) SARS-CoV-2-infected patients, respectively.

The activation profile analysis of CD4+ and CD8+ T cells showed a high frequency of CD95 expressing CD4+ and CD8+ T cells in patients with severe COVID-19 infection that persisted during the entire follow-up, suggesting strong T-cell activation. The patients with a mild disease showed a lower frequency of CD95-positive immune cells as compared with the four patients with severe clinical presentation in CD4+ and in CD8+ T cells (Fig. 2c, d). The expression of CD38 on CD4 and CD8 did not revealed evident differences (Fig. 2c, d). Cytotoxicity was further evaluated due to the high frequency of TEMRA T cells, defined by perforin in innate (NK) and adaptive immune cells (Fig. 3a). Blood from patients with severe disease exhibited a high frequency of perforin-expressing T cells at day 4 (58% in patient 1 and 57% in patient 2), which decreased at day 13 (13.3% and 14.1%, respectively) and day 18 (6.8% and 8.1%, respectively). In contrast, T cells from the patient with mild disease did not express perforin early after admission (d1). Both in severe and mild patients, all NK cells persistently expressed perforin, early after admission until the convalescent phase.

### Cytokine dynamics

The level of the pro-inflammatory cytokine IL-6 was found to be high at the time of admission in two out of four patients followed by a rapid decrease at day 8 with a slight increase at days 14–16 post admission in three out of four severe patients (Fig. 3b). A similar kinetic was observed also for IL-8, IL-1β and TNF-α showing an increase at late stage of disease. Mild patients showed a persisting low level of all the inflammatory mediators during all the course of infection.

The level of TGF-β was found to be higher in plasma from patients with severe disease, which decreased over time. In the early convalescent phases, around day 14 after admission, the levels of the inflammatory cytokines IL-1β, IL-8 and TNFα increased in parallel with a decrease in TGF-β (Fig. 3b) in all three patients.

### MDSC profiles

**Fig. 4:**
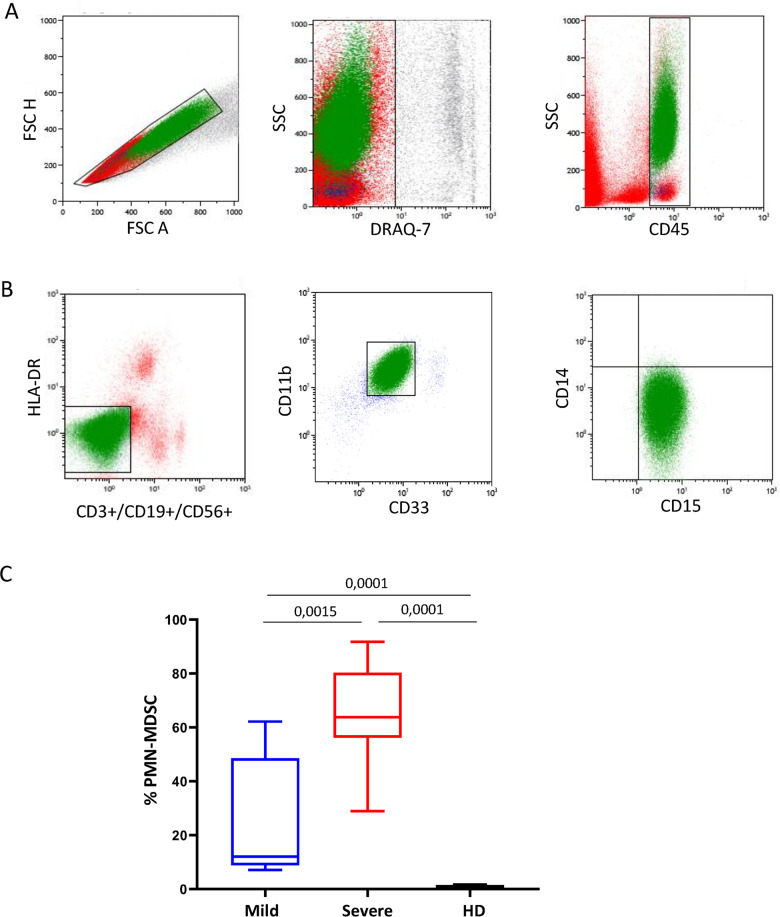
MDSC gating strategy and frequency in PBMC in SARS-CoV-2-infected patients. Myeloid-derived suppressor cells (MDSC) were identified in PBMCs by flow cytometry. **a**, **b** Representative plots of the adopted gating strategy to identify MDSC. Doublets were excluded in the FSC-H/FSC-A dot plot. In the leucocytes plot (SideScatter (SSC)/CD45), the CD45+ cells were gated (**a**), followed by gating on Lin-(CD3−CD19−CD56−)/HLA DRlow/− cell (**b**). Cells were then selected as CD3+CD11b+, and the expression of CD15+ and CD14− was evaluated (**b**). **c** Frequency of PMN-MDSC in nine mild (blue box), nine severe (red box) patients and in eight healthy donors (HD, black box). Results are shown as box and whiskers. The Mann–Whitney test was applied, and *p* < 0.05 was considered significant.

**Fig. 5:**
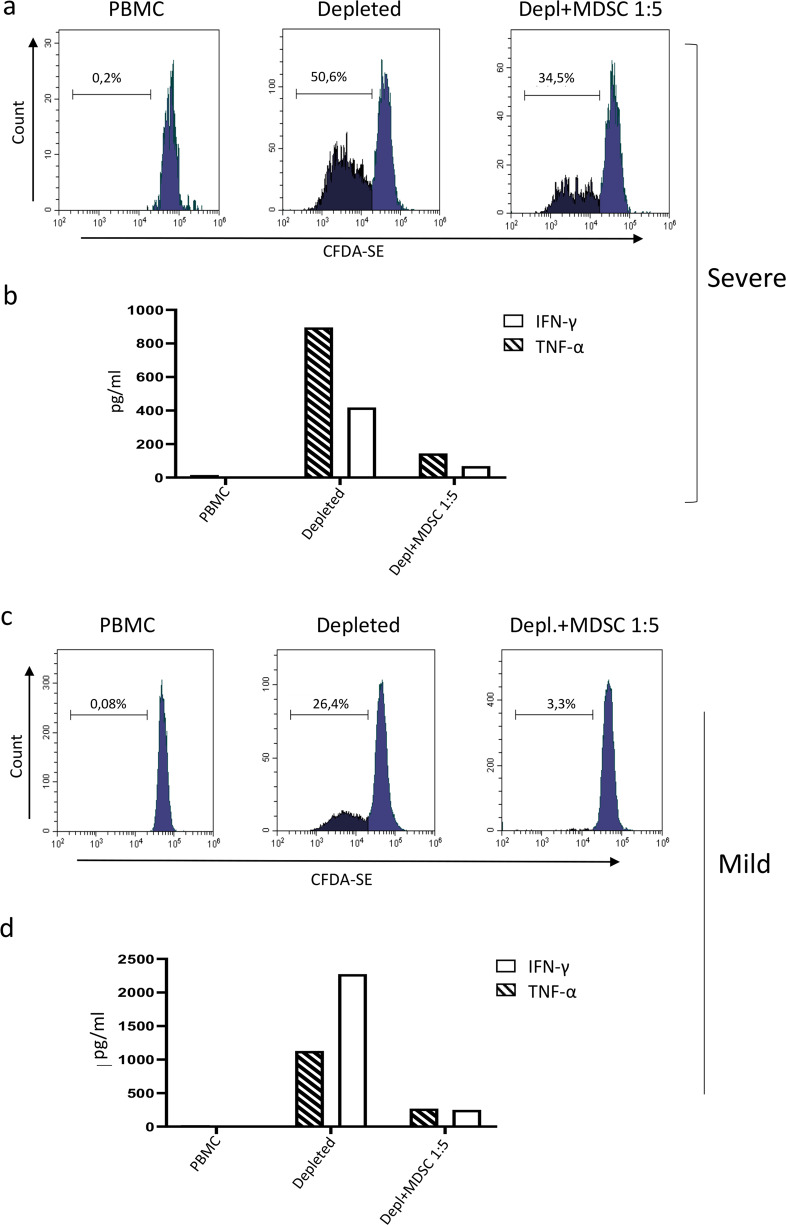
MDSC inhibition capability. MDSC suppressive capability was analyzed by testing T-cells proliferation and by cytokine quantification. PBMC and MDSC-depleted PBMC (depleted) from severe (**a**) and mild (**c**) SARS-CoV-2-infected patients were labelled with CFDA-SE. Purified PMN-MDSC were added to MDSC-depleted PBMCs at 1:5 ratio (depl.+MDSC 1:5). Staphylococcus enterotoxin B (SEB) was added to all conditions, and proliferation capability was analyzed after 4 days by flow cytometry. Cytokines were analyzed in supernatants of PBMC, PMN-MDSC-depleted PBMC and PMN-MDSC-depleted PBMC + purified PMN-MDSC (1:5) in severe (**b**) and mild (**d**) SARS-CoV-2-infected patients by automated ELISA after 48 h of culture.

We observed the presence of granulocytic cells after PBMC isolation by ficoll (Fig. 4a). Since it has been clearly demonstrated the stratification of the PMN-MDSC subset in the ficoll mononuclear fraction, we better characterize this cell population. We found that, indeed, these cells were HLA-DR− Lin−, CD33+, CD11b+, as described for MDSC (Fig. 4b). Moreover, they expressed CD15 and not CD14 (Fig. 4b), indicating the presence of the PMN-MDSC subset. We found a higher PMN-MDSC frequency in both COVID-19 groups than HD, and a higher frequency in severe than mild patients (Fig. 4c). MDSC are defined primarily by their immunosuppressive functions, which was confirmed by ex vivo isolated MDSCs, that were able to inhibit strongly T-cell proliferation (Fig. 5). PBMCs from patients with severe and mild COVID-19 infection failed to proliferate, reflecting an ex vivo suppressive environment. As MDSC were depleted from the patient’s PBMCs, immune cell proliferative capacity was restored, particularly in the patients with severe clinical presentation (from 0.2 to 50.6 % after MDSC depletion). When MDSC were added again, T-cell proliferation was strongly reduced from 50.6 to 34.5% (Fig. 5a–c). SEB—stimulation of immune cells showed that depletion of MDSCs resulted in a strong increase of IFN-γ and TNF-α production; vice versa, adding of MDSC decreased IFN-γ and TNF-α (Fig. 5b–d), confirming the biological activity of ex vivo isolated MDSCs in patients with severe COVID-19 infection.

**Fig. 6:**
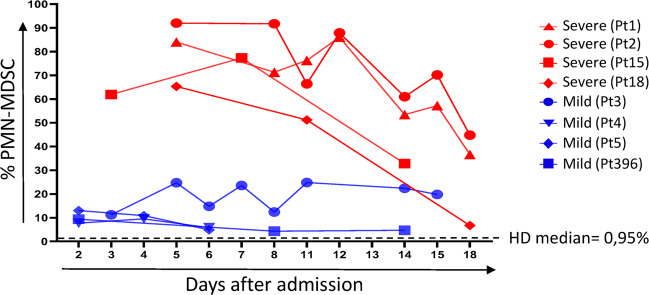
Kinetic of MDSC in SARS-CoV-2-infected patients. MDSC frequency was analyzed in PBMC from severe (Pt1, Pt2, Pt15 and Pt18) and mild (Pt3, Pt4, Pt5 and Pt396) COVID-19 patients at different time points. Dashed line: median of HD values.

The PMN-MDSC longitudinal analysis showed a substantial early increase in PMN-MDSC frequency in all patients with the severe COVID-19 presentation, up to 91.7% in patient 2 at day 5 after admission. In contrast, blood from all patients with mild COVID-19 infection exhibited a lower increase of MDSCs frequency, i.e., lower as compared with patients with severe disease presentation (Fig. 6). MDSCs decreased over time, yet were still found to be increased during the early convalescent phase as compared with the frequency of MDSCs in blood from mild individuals, whose MDSC frequency were stable over time (day 18, Fig. 6).

## Discussion

Most patients with COVID-19 show mild disease, whilst an estimated 20% progress to severe pneumonia, respiratory failure, and in 3, 2% cases, death [1]. There are several findings from the current study. First, lymphopenia, which has been found to be a common clinical feature in patients with COVID-19 [2], seen in our four cases with severe disease. Whilst lymphopenia may turn out to be a clinically relevant finding associated with disease severity and mortality [3], we did not identify any specific T-cell subsets to be preferentially affected. Second, a massive expansion of functional MDSCs (reaching up to 90% of total PBMCs) in severe COVID-19 disease associated with a strong activation of the immune system. Third, in the later convalescent phase of disease severe cases, the reduction of MDSC frequency was associated with an increase of pro-inflammatory cytokines along with reduction of TGF-β, indicating a relationship between MDSC expansion and reduction of inflammation. MDSC expansion is a common response to most inflammatory processes, and the functions of MDSCs are highly dependent on the inflammatory environment. Fourth, our findings of T-cell activation, differentiation and cytotoxic profile indicates that hyperactivation of both the innate and adaptive immune arms of the immune system may be a hallmark of severe disease and play a role in COVID-19 pathogenesis. This is supported by findings seen in the four patients with severe disease: (1) increased frequency of differentiated T cells (both CD4+ and CD8+ T cells) with a reduction in precursor T cells and a parallel increase of EM CD4+ and TEMRA CD8+ T cells; (2) persistence of highly activated T cells; (3) a very high frequency of cytotoxic T and NK cells; and (4) a higher inflammatory response (IL-6).

The expansion of MDSCs can be induced by several factors (e.g., plasma elevation of prostaglandins, M-CSF, G-CSF, GM-CSF, IL-6 and VEGF) [10]. It is therefore possible that MDSC expansion in patients with severe disease could be driven by hyperactivation of the immune system, excessive inflammatory mediators and growth factors as recently described by Huang et al. in patients with COVID-19 infection admitted to the ICU [2]. According to this model, the patient with mild disease showed an undisturbed T-cell differentiation profile and lower frequencies of immune cells exhibiting an activation/cytotoxic profile. This was paralleled by lower frequencies of MDSC compared with patients with severe disease. These MDSCs appear to be highly functional, irrespective of the disease severity; MDSCs both from patients with severe and mild symptoms strongly suppressed T-cell proliferation and cytokine production. The fact that ex vivo MDSC depletion restored T-cell functions, confirmed their potential suppression function in vivo. Accordingly, high IL-6 levels have been recently associated with fatal COVID-19 infection [11], suggesting that immune suppression possibly mediated by expanded MDSCs could be highly beneficial in reducing inflammation and lung damage induced by hyperactivated cytotoxic cells [12]. On the other hand, we could not rule out the possible detrimental role of expanded MDSC in inhibiting a protective cytotoxic immune response.

The suppressive functions of MDSCs are mediated by several molecular pathways, including inducible nitric oxide synthetase, arginase-1, NADPH oxidase and TGF-β [10]. MDSCs can inhibit several immune cell functions such as T-cell proliferation and activation [13–16], cytokine production by macrophages [17] and NK-cell function [18]. They are also involved in the induction of T regulatory cells [19], playing a pivotal role in regulating immune response. Furthermore, a correlation between Th17 and MDSC has been described in several disease settings [20, 21]. High level of plasmatic IL-17 has been reported during COVID-19 [22], however, its correlation with MDSC remains to be elucidated.

MDSC expansion is a response to most inflammatory processes in order to curb excessive, and potentially harmful, immune responses. Several conditions associated with excessive inflammation result in aberrant myelopoiesis, characterized by accumulation of myeloid cells with regulatory functions: MDSC are a heterogeneous cell population at different stages of differentiation, comprising myeloid cell progenitors and mature cells [23] that expand under certain pathological conditions such as cancer, inflammatory diseases and autoimmune disorders [13–15, 24, 25]. Two major MDSC subsets have been identified in humans, the monocytic-MDSC and PMN-MDSC subsets, expressing low level of HLA-DR, myeloid markers as CD11b and CD33, and CD14 or CD15, respectively [13]. Patients experiencing viral infections, other than COVID-19, were found to have high levels of MDSCs. For example, in patients with dengue infection the expansion of MDSC correlates with dengue virus viral load, clinical severity score and a decrease in fever [26]. In general, MDSC may reduce immune activation in acute infection, thus dampening immune pathogenesis as shown in a sepsis model [27]. MDSCs were first studied in patients with cancer, yet were found to modulate immune responses in infectious diseases as well as in other inflammatory conditions [13–15, 24, 25]. Although clinical treatments have been developed to downregulate MDSCs in patients with cancer in order to restore productive anti-tumour immune responses [25], recent studies suggest that MDSCs may even play a positive role in acute inflammatory processes, by dampening potential harmful, non-protective immune responses [27, 28]. For instance, MDSC expansion during dengue infection downregulates the inflammatory response and subsequently immune-mediated pathology [26].

In sepsis, MDSC seems to play a dual role depending on the disease progression: they are beneficial by reducing inflammation during the early stages of sepsis, thus protecting from early organ dysfunction. At a later stage of the disease, MDSCs can be detrimental by amplifying long-term immunosuppression [27–29]. Our data suggest a protective role of MDSC expanded early during the acute phase of the COVID-19 reducing T-cell hyperactivation and inflammation but, on the other hand, they can dramatically dampen the protective immune response. This finding is in line with previous observations in bacterial or fungal infections, namely, that a strong pro-inflammatory response is followed by a counter-regulatory process that has been coined as “immune paralysis” [30]. This information is clinically meaningful, since restoration of immune competence during “immune paralysis” has been achieved, for example, with IFN-gamma [31] or with IL-7 [32] and found to be associated with improved clinical outcome.

The dynamic nature of the immune response during COVID-19 infection, described in this report, underlines that longitudinal sampling is needed in order to define the best time point for therapeutic intervention(s)—depending on the status of the hyper-inflammatory or hypo-inflammatory disease process. Although including a limited number of patients, this study shows a huge expansion of MDSC in severe COVID-19 associated to a reduction of inflammatory mediators. The protective role of MDSC needs to be further evaluated and confirmed in studies with larger cohorts of patients with severe COVID-19 disease in order to tailor specific immune-mediated interventions and/or define a possible role of MDSCs as markers of disease activity.

## Data Availability

The datasets generated during and/or analyzed during the current study are available from the corresponding author on reasonable request.
